# Hospital and Institutional News

**Published:** 1919-03-01

**Authors:** 


### HOSPITAL AND INSTITUTIONAL NEWS.

### THE BRITISH PHARMACOPŒIA.

THE Executive Council of the General Medical Council has considered the advisability of withdrawing the temporary alterations in the *British Pharmacopœia*, arising out of the scarcity during the war of sugar, glycerine and certain oils and fats, and adopted the following resolution:—"That the Executive Committee on behalf of the Council order and direct that on and after April 30, 1919, the *British Pharmacopœia*, 1914, shall be altered and amended by revoking and withdrawing the alterations and amendments made and published in the *Gazettes* of July 27, 1917, and March 29, 1918; and that formal intimation of such revocation and withdrawal be published according to law in the *Gazettes* of London, Edinburgh and Dublin on April 30, 1919."

### LOOK AFTER THE NATION'S TEETH.

A BRASS plate, plenty of assurance and an easy conscience are the main assets needed by the quack who wishes to set up a "dental surgery." Failures in every other walk in life find it an easy way to make a living, and providing the pirate with the forceps does not kill a patient, break his jaw, or poison him with cocaine, he can carry on with impunity. It did not need a Government Committee to disclose the iniquities that are carried on in the name of dentistry, or the injustice to those with diplomas of permitting a host of men without experience to prey upon the, to a large extent, unsuspecting public. But the Departmental Committee has now reported, and it recommends, among other things, (1) That a public dental service be established at once, which should be free and entrusted to the public health authorities. (2) That the Board of Education should obtain quickly, dental treatment for the 6,000,000 children in the elementary schools. (3) That the cost of a dentistry diploma should be reduced, and the three years' study reduced to the minimum. (4) That all unqualified practice should be made illegal, with penalties. As a nation, perhaps we have the worst teeth in the world, and Government action is long overdue. The matter has such a vital bearing on the public health that it might very well find a place in Dr. Addison's Bill.

### THE SALE OF COCAINE AND MORPHINE.

THE recent disclosures in a Coroner's Court demonstrate, in the clearest way, that in spite of all precautions it is possible to buy cocaine and morphine in London for illicit purposes. We say "all" precautions, for, short of the absolute prohibition of the importation of cocaine into this country or its manufacture here, it is impossible to make the present restrictions more secure. No Act of Parliament will kill all the black sheep, and even if it were made a criminal offence for any one to be in the possession of cocaine, no Act of Parliament could make its possession impossible. Moreover, it would be impolitic to prohibit the proper use of an invaluable drug in order to protect here and there some one from abusing it. What we are anxious about is that the present restrictions should not be relaxed. With all the talk about D.O.R.A. dissolving herself, there is a danger that some of the precautions against the distribution of narcotics will be removed, but we sincerely hope that one of the memories that D.O.R.A. will leave behind will be the permanent embodiment in an Act of Parliament of the regulations against the sale of cocaine and morphine to unauthorised persons. There is nothing in pharmacy legislation anything like sufficiently drastic to keep the sale of these habit-forming drugs in proper bounds. For instance, there is nothing in these Acts to prevent any one from buying a million doses of cocaine every day: if he cannot sign his name in the chemist's "poison book," presumably he can make a cross. Even though a duly registered chemist omits a dozen times to comply with the mechanical formalities required by the Pharmacy Acts, and is fined a dozen times for these omissions, he may still continue the ghoulish game—the Pharmaceutical Society has no power to strike him off the Chemists' Register. Under the Defence of the Realm Act a chemist who sells cocaine improperly may be prohibited from dealing in it in future—one was inhibited the other day. We hope that when the Defence of the Realm Act is a thing of the past the same power will be vested either in the Home Office or the Pharmaceutical Society.

### DISABLED MEN IN AMERICA.

IN a statement entitled "Facts of Interest to the Disabled Soldier and Sailor" and approved by the various Government departments concerned, the American Red Cross Institute for disabled men outlines a scheme for the restoration of the injured to self-support. Some of their points indicate both thoroughness and liberality. After discharge from the service, if the man is disabled to any considerable degree—so as to be entitled to compensation for disability—he is offered training for a skilled job in which his injury will not prevent his earning good wages. Compensation will not be reduced or in any way affected by what he is able to earn. Training after discharge will be provided at Government expense, and during the course of training, in order that he may have no financial worries, he will receive either the same pay as during his last month in the service or his compensation for disability, whichever is the larger. His family will continue to receive the same allotment and allowances as when he was in service. When training is completed the Government will find him desirable work. Employers are giving careful thought to the sections in which his services can be used to the best advantage; they realise that what he wants is not charity but the opportunity of self-support. Labour unions are deciding in which ways disabled men may best be replaced in their trades, and are prepared to assist the readjustment to the greatest possible degree. We hope that these commendable plans will achieve the success they deserve.

### NATIONAL SERVICE MINISTRY.

According to a statement recently issued, the Medical Department of the Ministry of National Service will be transferred to the Ministry of Pensions, and arrangements are now in progress to this effect. The medical and secretariat staff affected by this change, both at the headquarters of the Ministry of National Service and at the offices in the regions and areas of the Ministry, will continue to carry out their duties as at present, and officials will receive notice in due course of all arrangements made for their transfer. There is an exception, however, in that the offices of the Chief Commissioner of Medical Services and the branch (M. 4) dealing with demobilisation of medical and dental officers on service with the armed forces of the Crown will remain with the Ministry of National Service.

### WHISKY CURE FOR INFLUENZA.

There is a consensus of opinion, especially medical opinion, that stimulants, whisky in particular, are a preventive of influenza. The filter-passing virus may elude scientific vigilance, but by all accounts it stands a poor chance against the penetrating potency of distilled spirits. In face of this even the Liquor Control Board, which hitherto has hardened its heart against both threat and appeal, has yielded, and whisky, brandy, and gin are now to be available in larger quantities for those who have faith in their fortifying qualities. The teetotal propagandist, however, is not happy. He looks upon this concession as a victory for the liquor interests, and a score for those who favour a return to free trade in intoxicants. Dr. John Clarke, of Woolwich, makes a suggestion which, if adopted, would at any rate soothe this class of objector, even if it did not satisfy him. The suggestion is that whisky and brandy should be placed at every police station throughout the land, and distributed to the sick under practitioners' certificates at all reasonable hours and at reasonable prices. There could be no public-house atmosphere about this. But while the teetotaller might be mollified, it is fairly certain that "the trade" would discover a volume of objections. *Apropos* of the whisky cure for influenza, it is curious that the State Government of Victoria has, as a protective measure against the epidemic, ordered the closing of all hotels within a fifteen-mile radius of Melbourne. Victoria has taken the wrong turning. However, the whisky cure has burst upon the Motherland with the suddenness of a revelation, and the good news must be given time to penetrate to our Australian kindred. When it does we can make a shrewd guess who will be among the first converts.

### JUSTICE TO IRELAND.

It has been the subject of comment that the provisions of the Ministry of Health Bill, which are expected to do so much for Great Britain, do not extend to Ireland, where, it is stated, the incidence of sickness is one in two. This matter was referred to by Mr. Macpherson, Secretary of Ireland, in his reply to a deputation on health affairs which waited upon him in Dublin. He pointed out that centralisation, which was the main feature of Dr. Addison's measure, already to a large extent exists in Ireland by the powers given to the Local Government Board. At the same time it was their business to see that any legislative advantage to England should be extended to Ireland in the new Bill, or that a Bill like it should be passed for the good of Ireland. One could not be blind, he added, to the fact that the health of the children in Ireland was not good, and the medical inspection of schools and school children which had proved so beneficial in other parts of the United Kingdom would be introduced by means of a special Bill.

### PROBLEM OF LIMBLESS SOLDIERS.

The appointment in the House of Commons of a Committee to inquire into the future of limbless soldiers is necessary and satisfactory in principle, but it is a pity that the medical element is not more in evidence. The construction and fitting of artificial limbs, apart from the surgical and medical aspects of these cases, call for a very specialised knowledge. As is remarked in the *Times*, it is unfortunate, when this country can claim in Sir Robert Jones one of the greatest orthopædic surgeons in the world and the founder of a regular school of orthopædic surgery, that the advantages of advice from some of his distinguished followers have not been utilised. The problem is one in which expert professional opinion should be paramount and followed, rather than led, by ministerial action. We hope the arrangement is not permanent and that a more rational policy will have been adopted by the time any scheme is put into operation.

### TREASURY GRANT TO VOLUNTARY HOSPITAL.

It was announced in the annual report of the governors of the Royal Devon and Exeter Hospital that to enable the institution to deal with cases of discharged sailors and soldiers a Treasury grant of £2,000 had been made towards the cost of additional accommodation. This sum is to form the nucleus of a fund for extension, and Lady Wills and the President of the hospital have each promised £1,000 towards the same object. It was not stated what will be the amount of the capitation grant paid in respect of the pensioners, but presumably it will be the same as elsewhere, namely, 6s. a day. The bargain with the Treasury is a good one, because, in view of the fact that the pensioner patient will sooner or later no longer require treatment, the full value of the extra accommodation will accrue to the hospital for unrestricted use to further its ordinary objects.

### NO INFLUENZA CASES NEED APPLY.

At the annual meeting of the York Workpeople's Hospital Fund, Mr. Frederick Neden, secretary of the York County Hospital, made a startling statement. He said that the decreased number of civilians in the hospital was due to the fact that two or three wards had been closed owing to the influenza epidemic. There seemed to be signs of a fresh outbreak, and the Hospital Committee had taken action at its last meeting by deciding that no cases of epidemic pneumonia should be admitted. This, Mr. Neden explained, was not by any means on account of the staff shirking their duties—they were quite willing to take all the risks of attending such cases. The risks were very heavy, and more than twenty members of the staff were incapacitated by the epidemic. One had died, and he thought the hospital had done its share in dealing with the epidemic when it broke out last October. So in respect of York voluntary hospital help in the present hour of need is no longer available. We notice that York County Hospital is appealing for £4,000 to pay off debt.

### ENDOWMENT OF FAMILIES.

Mr. Emile Burns has placed before the National Birth-rate Commission the views of the Family Endowment Committee on the birth-rate problem. From a financial aspect they are rather startling. To encourage large families his Committee proposes that the State should provide a regular weekly income to families with children under fifteen on the following basis: Mother eight weeks before confinement and until her youngest child is five, 12s. 6d. per week; each child from birth until school age—for the first child 5s. per week, and for the second and subsequent children 3s. 6d. per week. The total cost would be £240,000,000 per annum. As the population increases under this benevolent scheme of subsidies presumably the total would grow higher and higher. The Family Endowment Committee holds lofty ideals, and clearly it does not permit practical politics to stand in its way.

### TEACHING THE ADOLESCENT.

Like Sir Robert Armstrong-Jones, Lecturer on Psychological Medicine to St. Bartholomew's Hospital, the Bishop of Birmingham is of opinion that in the matter of venereal disease the State should step in where parents and religious teachers apparently fear to tread, and teach adolescents the dangers that lie in wait for them. In his preface to the annual report of the National Council of Public Morals, the Bishop urges that all on the threshold of adult life should be warned, shown the best, and stirred to higher ideals. This, he thinks, could be accomplished by the introduction of sex education into schools. In the past, when the State lost control of the young about the age of fourteen, there were difficulties in the way of carrying out this ideal, but now, when continuation schools are proposed to be compulsory up till sixteen, the path is made much smoother for the introduction of instruction on this subject of health and morals.

### INDEPENDENT CAMBERWELL.

There has always been a spirit of independence in Camberwell, and it is once again being displayed in connection with smallpox. The London County Council asks Camberwell to fall into line with other Councils which have provided shelters for persons who have been in contact with smallpox cases. The Camberwell medical officer of health, however, whilst admitting the value of surveillance over contacts, reiterates his view that it is better to keep contacts under observation at their own homes, rather than remove them at great expense, trouble, and inconvenience, to some other place or shelter. The issue, says the medical officer, is this: are we, at considerable expense, to do away with a system that we have found to work perfectly well long before the L.C.C. thought it necessary to call attention to the desirability of removal to special premises?

### MILK SUPPLY WITHOUT RETAILERS.

Milk retailers are a useful body, but Saltcoats has proved that they are not indispensable. The local distributors in that place, being dissatisfied with the retail prices fixed by the Food Control Committee, decided to strike, regardless of the health and convenience of the community. Nothing dismayed, the Committee set to work to organise the distribution of milk, and the farmers proving friendly the scheme thus set on foot has worked well for some months and is now on a permanent basis. When the Food Control Committee goes out of business, the municipal authority will "carry on." Milk retailers in other places, when they feel tempted to push their claims to the bitter end, should ponder the fate of the traders of Saltcoats.

### WEST SUFFOLK GENERAL HOSPITAL

Like many other Committees, the Committee of the West Suffolk General Hospital, Bury St. Edmund's, betrays anxiety in its annual report as to the future. With the expenditure now £7,600, against £3,713 before the war, it is wondering how ends can be made to meet in normal times when civilian patients replace wounded soldiers, for whom the War Office in 1918 paid £2,860. Happily, the income shows healthy elasticity in all its branches, and one new feature in particular promises future expansion. This consists of a Ladies' Association for the collection of subscriptions in the various parishes of the county from those who have not hitherto supported the Hospital, also for obtaining money by entertainments, and so on. By these means £337 was raised last year, a sum which on this occasion is to be applied to some special need of the institution as it is not required to meet current expenses. The Committee is gratified at this initial effort, and hopes it will grow, as there is every promise of difficult times ahead.

### GUY'S RESIDENTIAL COLLEGE SCHEME.

Suggestions as to the form Guy's Hospital memorial should take are not coming in so freely as the Editor of the *Gazette* would like. So in the current issue the Editor steps boldly into the breach with a concrete proposal. It is pointed out that, owing to the large number of junior students in residence, it is often impossible for those holding ward appointments to obtain rooms in the Residential College, and it is urged that the whole of the accommodation—for about sixty—should be available for these. "Is it not a practical suggestion," it is asked, "that an establishment on the lines of the College should be built away from the present site—say close to the athletic ground at Honor Oak Park, or even in some more countryfied spot?" The advantages of such an establishment to men not holding ward appointments are dwelt upon, particularly its nearness to the athletic centre and the possibility of running it on more economical lines than the present College. The proposal is submitted to the consideration of the War Memorial Committee, and, at the same time, criticisms and amplifications of the scheme are invited.

### THE RED CROSS IN PEACE.

When the war burst upon us the Red Cross Societies of the Allies were comparatively puny organisations. They have grown into powerful bodies, with every means at their disposal to reduce the sum of human suffering and pain. Thoughtful people are saying it would be deplorable if all these engines for good, because the war is over, were consigned to the scrap-heap when the demand for such help is greater, perhaps, than ever it was before. Plans are therefore being set afoot to enable the beneficent workers to tackle the problems of peace as they fought the miseries of war. A convention of the Red Cross Societies of the world is to meet at Geneva thirty days after the declaration of peace, "to formulate and propose an extended programme of Red Cross activities in the interest of humanity." From this is expected to issue an international organisation, through which the peoples of the world may co-operate in fostering the study of human disease, promoting sound measures for public health and sanitation, the welfare of children and mothers, the education and training of nurses, and the care and prevention of tuberculosis, venereal disease, malaria, and other chronic or infectious diseases; and providing measures for handling problems of world relief in emergencies such as fire, famine, and pestilence. It is a monumental programme. Many of these problems are already being tackled by the Governments on both sides of the Atlantic. But a world-embracing movement would assist, and not retard, the special efforts of individual nations.

### SPOILING THE SHIP FOR A HA'PORTH OF TAR.

A deputation of the Monmouthshire Insurance Committee recently visited Highfield Hospital, one of the Sanatoria of the Welsh National Memorial Association, and the report they made on the internal arrangements was far from being satisfactory. The hospital stands in an elevated position in one of the most healthy spots in Monmouthshire, having the full advantage of the sun from its rise to its setting, together with the purest air both from the sea and the country. There is no complaint about the hospital, but as to the management there has been much adverse criticism, chiefly on the part of the patients, which the insurance visitors evidently consider is not without foundation. The bacon is said to be uneatable, because of its poor and salty character, tinned milk is supplied instead of cow's milk, and if the monotony of the margarine were broken by an occasional supply of jam or cheese the absence of butter would be better tolerated. Enamel drinking-cups, more or less chipped, are used instead of china mugs. It is good news to hear that the Insurance Committee are taking such steps as should lead to a better state of affairs.

### VETERAN INSTITUTIONAL WORKER.

The death has just occurred in his 92nd year of Mr. Charles Holmes, who retired in 1895 from the Metropolitan Convalescent Institution after forty-two years' service. For the greater part of this period he held the position of Secretary. Upon his retirement he was elected to a seat upon the Board of Management, and his attendances at the meetings were regular until a few years ago, when the difficulties attending the journeys to town for this purpose became too much for him. Although so very advanced in years, he kept his keenness of intellect until the very last, and we understand that his end came quite peacefully and without any previous suffering. He formed a very interesting link with the Metropolitan Institution with which he became connected soon after its establishment in 1840, and its present popularity and eminence are due, in a measure, to his long and energetic service.

### TRANSVAAL INSPECTOR OF HOSPITALS.

The Transvaal Provincial Administration has created a new post, that of Inspector of Hospitals. This is a result of the recommendation of the Hospital Committees' Conference urging the appointment of a Director of Hospitals. The Transvaal Executive, while not carrying out the recommendation of the Conference in its entirety, has thus partly met it. The new office has been accepted by Dr. McKenzie, with the concurrence of the Johannesburg Hospital authorities. Dr. McKenzie, while continuing his present Johannesburg duties, will (1) periodically render such assistance as is possible to committees that require it; (2) advise the Provincial Administration on technical matters; (3) and report to the Administrator on his periodical visits to institutions.

### THIS WEEK'S DRUG MARKET.

There is perhaps a slightly improved tone in business this week in a better demand for several products including camphor, peppermint oil, and menthol. Of these camphor is substantially dearer; peppermint oil and menthol are also dearer. Apart from these few articles, however, such price changes as there be are in a downward direction. There is more demand again for eucalyptus oil, but with fresh supplies arriving to add to existing stocks, prices are inclined to move downwards. Several of the synthetic drugs are again lower as, for instance, aspirin, salicylic acid, benzoic acid, and phenacetin, while sulphonal at last shows signs of weakness. Hexamine, on the other hand, tends upwards in price, and saccharin would appear to have touched the lowest level.

